# Reports on Hospitals of the United Kingdom

**Published:** 1914-01-24

**Authors:** Henry Burdett


### REPORTS ON
### Hospitals of the United Kingdom.

By SIR HENRY BURDETT, K.C.B., K.C.V.O.

SERIES III.

### HUNTINGDON COUNTY HOSPITAL.

This hospital was established in 1853 and enlarged in 1863. Structural improvements were made in 1897 and 1903, and the children's wing was opened in 1912. This hospital has accommodation for some sixty patients. Its wards have no cross ventilation, though the large ones have the advantage of a verandah running the whole length. All the wards are on the first floor, but the verandahs are not wide enough to allow the beds to be placed at right angles to the main building. The latter should be reconstructed to permit of this being done. Several of the wards have ventilators some eighteen inches in diameter placed in the ceilings, which communicate with the outer air through the roof. These ventilators have no exhaust fans, which could readily be fixed at their bases and worked by electricity. At present they may act, we imagine, more as disseminators of infection than as ventilators, and it is possible that the sore throats, which we learned were fairly frequent among the patients, may be due to this cause, as has been the case elsewhere. It might be best to close up these ventilators and the openings in the ceilings altogether.

### Some Special Features.

This is an old-fashioned, quiet, placidly-working institution which bears upon it few marks of the great changes which have taken place in larger and more up-to-date hospitals. The housekeeping is economically done, the nursing is good, though the staff is none too large when the beds are fully occupied, and the accommodation for the nursing staff is, on the whole, reasonably good. In 1912, as a memorial to the late King Edward VII., a new wing on the western side of the main building was erected to provide accommodation for the nursing staff. The more important surgical work which arises in the course of the year is undertaken by the consulting surgeon, Dr. J. Griffiths, of Cambridge, only minor cases being dealt with by the honorary medical staff. The operation theatre is well lighted and fitted, and fulfils every requirement.

### Too Much Economy?

The number of out-patients seems large having regard to the size of the institution and the population served. Speaking generally, we should say that the institution is comfortably managed from the patients' point of view, but there are certain directions in which improvements could be effected. Its equipment is not so ample as it might be owing to the so-called strict economy enforced by the committee. They might, for instance, usefully spend a little money on the equipment of the kitchen and its adjuncts, which are not attractive at present. The so-called laundry department is contemptible as it stands, and should be taken in hand and put in order without delay. The mortuary, too, the door of which is kept constantly open, was dirty and resembled a neglected dust receptacle, which reflects no credit on the hospital, nor does it do justice to the scientific spirit that no doubt animates the medical staff. The *post-mortem* room should be taken in hand at once, thoroughly cleansed, and put and kept in order for use. This will probably mean some reconstruction, as from false notions of economy the only approach left to the electrical machinery is through the *post-mortem* room door, which is kept wide open. We refrain from further comment; the moral is obvious.

### Public-spirited Nurses.

We observed that the nurses, with great public spirit, had organised an entertainment which was to be given on the night of Saturday, October 25, the day of our visit. The proceeds of these entertainments are about £20, and we venture to say that instead of being placed to the credit of the general fund of the hospital, they might properly be handed to the matron to enable her to expend the money upon such further equipments of the wards as would facilitate the work of the nurses and the comfort of the patients. In front of the hospital is a beautiful lawn, the beauty of which is enhanced by two fine cedars in full vigour and of great charm. It has, we believe, one of the most attractive hospital tennis courts in the country.

### BURY ST. EDMUND'S HOSPITAL.

This hospital was erected in 1824, and, with the exception of certain improvements and additions, the buildings appear to be pretty much what they were originally. Recently the wards have been tiled, new verandahs have been added, and more accommodation has been provided, thanks to the generosity of the governors, through which each nurse has a separate room. We understand the lavatories and bathrooms were tiled at the same time as the wards. The baths might well have been replaced by new ones of Doulton's enamelled steel of the best pattern, seeing that the present painted baths can never be made to look really smart, even when newly painted. The whole of the hospital is well kept, so far as it is possible to keep well an old hospital of this type, but the sisters and nurses should be encouraged by the provision of better accommodation for the bedpans and other necessaries in the lavatories, with several other things which would tend to smarten up the wards and provide accommodation for stores of various kinds. We noticed that recently a provision larder has been supplied on each floor in which the food is kept. This new departure must have been a marked improvement on the old arrangements, and might be generally adopted.

### Underpaid Sisters and Good Nurses.

We visited this hospital in the afternoon of October 24, 1913. Though we missed the matron, we were very courteously received by Dr. Burke, house surgeon, and a sister from the children's ward, who took infinite pains to enable us to see everything in the hospital. For its size and type we regard it as efficient, but we think that the sisters should have more helpers, for they have upstair and downstair wards containing some thirty-eight beds, in addition to the verandahs. They have also, the one to take charge of the children's ward, and the other of the operation theatre and casualty department, which involve onerous duties, and so much work must at times devolve upon each of them. They should be paid not less than £35 a year each, with a bonus of £10 extra so long as they have these extra duties. These two sisters must be women of character, experience, and ability. They should receive a salary commensurate with the work and responsibility which devolve upon them. Speaking of the nurses generally, we should say from the general orderliness and smartness of the wards that they are a good type of woman, who carry out their work cheerfully and well, despite the difficulties which must necessarily attend service in an old building, where the equipment errs on the side of incompleteness rather than on that of fulness.

### The Chapel and Mortuary.

The hospital has a chapel where the nursing staff assemble every evening for a short service, and where the patients attend on Sundays, and occasionally on week-days too. The mortuary accommodation is simple, adequate, and well equipped. The *post-mortem* section has recently been entirely renewed, while the view room has been simply decorated and made suitable to its purpose.

### A Valuable Hospital.

The hospital is, we are glad to learn, valued by the people of Bury, who display a considerable interest in it. The accounts show that the support extended to it is liberal, and nearly sufficient to make both ends meet each year. The Report contains abstracts from the old accounts from March 28, 1826, to December 31, 1912. It would appear from these accounts that the total sum expended upon the upkeep and maintenance during the whole period amounted to £212,668. The voluntary gifts to the hospital during the period were about £154,000, and the original cost of the buildings was less than £12,000. The investment funds appear from this abstract to amount to upwards of £30,000.

### Well-deserved Congratulations.

We were very sorry not to have had the pleasure of meeting the matron, Miss Brown, and the committee, to congratulate them on the condition in which the hospital was at the time of our visit. We expect that the excellent verandahs, which are a great addition to this hospital in usefulness and importance, will be made increasing use of during the greater part of each year, as the people and the staff become thoroughly accustomed to outdoor treatment.

**Addenbrooke's Hospital, Cambridge, will be reported on next week.**

